# Alpha-ketoglutarate ameliorates abdominal aortic aneurysm via inhibiting PXDN/HOCL/ERK signaling pathways

**DOI:** 10.1186/s12967-022-03659-2

**Published:** 2022-10-08

**Authors:** Junjun Liu, Mingyuan Liu, Jiaxuan Feng, Hongqiao Zhu, Jianlie Wu, Heng Zhang, Shun Xiao, Zaiping Jing, Jian Zhou, Haitao Niu, Mingjin Guo

**Affiliations:** 1 Department of Vascular Surgery, The Affiliated Hospital of Qingdao University, Qingdao, 266000 Shandong China; 2 Department of Vascular Surgery, Beijing Friendship Hospital，Capital Medical University, Beijing, China; 3 Department of Vascular Surgery, The First Affiliated Hospital of the Navy Medical University, Shanghai, China; 4 Department of Urology Surgery, The Affiliated Hospital of Qingdao University, Qingdao, 266000 Shandong China; 5 Intervention Center, Shanghai General Hospital, Affiliated to Medical School of Shanghai Jiaotong University, Shanghai, China

**Keywords:** Abdominal aortic aneurysm, Alpha-ketoglutarate, ROS, PXDN, HOCL, ERK1/2

## Abstract

Abdominal aortic aneurysm (AAA) represents the serious vascular degenerative disorder, which causes high incidence and mortality. Alpha-ketoglutarate (AKG), a crucial metabolite in the tricarboxylic acid (TCA) cycle, has been reported to exert significant actions on the oxidative stress and inflammation. However, its role in AAA still remains elusive. Herein, we examined the effects of AKG on the formation of AAA. The study established an elastase-induced mouse abdominal aortic aneurysms model as well as a TNF-α-mediated vascular smooth muscle cells (VSMCs) model, respectively. We displayed that AKG pre-treatment remarkably prevented aneurysmal dilation assessed by diameter and volume and reduced aortic rupture. In addition, it was also observed that AKG treatment suppressed the development of AAA by attenuating the macrophage infiltration, elastin degradation and collagen fibers remodeling. In vitro, AKG potently decreased TNF-α-induced inflammatory cytokines overproduction, more apoptotic cells and excessive superoxide. Mechanistically, we discovered that upregulation of vpo1 in AAA was significantly suppressed by AKG treatment. By exploring the RNA-seq data, we found that AKG ameliorates AAA mostly though inhibiting oxidative stress and the inflammatory response. PXDN overexpression neutralized the inhibitory effects of AKG on ROS generation and inflammatory reaction in MOVAS. Furthermore, AKG treatment suppressed the expression of p-ERK1/2, 3-Cl Tyr in vivo and in vitro. ERK activator disrupted the protective of AKG on TNF-α-induced VSMCs phenotypic switch. Conclusively, AKG can serve as a beneficial therapy for AAA through regulating PXDN/HOCL/ERK signaling pathways.

**Supplementary Information:**

The online version contains supplementary material available at 10.1186/s12967-022-03659-2.

## Introduction

Abdominal aortic aneurysm (AAA) represents a degenerative disorder that irreversibly affects human health, and it is associated with a high mortality once aortic rupture [1]. AAA has the feature of progressive local dilatation of abdominal aorta with the diameter of more than 30 mm [2]. Its risk factors are dyslipidaemia, hypertension, smoking, male gender, family history and atherosclerosis [3]. At present, AAA can be mainly treated by invasive endovascular stent graft therapy or open surgery, while there have been no safe and effective medication available for disease management [2].

AAA is an inflammatory vascular disease. Vascular smooth muscle cell (VSMC) apoptosis, maladaptive aortic wall remodeling and oxidative stress (OS) are pathological features of the progression of AAA formation [4]. Chronic inflammation and excessive reactive oxygen species have long been recognized to be the main causes of AAA. In the initial phases of AAA diseases, infiltration of inflammatory cells into aortic wall can be observed, along with increased production of proinflammatory mediators. Then, infiltrating cells trigger the inflammatory response and promote VSMCs phenotypic switching. Furthermore, inflammatory cells secrete extracellular matrix (ECM) degrading enzymes to destroy aortic wall matrix during vascular remodeling [5–7]. In addition, vascular oxidative damage is among the risk factors for early AAA. Genetic and pharmacological inhibition of excessive ROS production significantly reduced AAA diseases [8].

Alpha-ketoglutarate (αKG) accounts for a critical metabolic intermediate produced from tricarboxylic acid (TCA) cycle for maintaining energy homeostasis [9]. AKG has previously suggested to have an essential effect on anti-aging [10], anti-inflammatory response [11] and anti-tumor effects [12]. Not only that, as an antioxidant, AKG is also important for diverse oxidative reactions [9, 13]. These pleiotropic functions of AKG have a beneficial effect on the treatment of cardiovascular disease including enhancing myocardial energy production, reducing the incidence of myocardial ischemia and preventing cardiac remodeling [9, 14]. According to the results, AKG possibly has certain impact on inhibiting AAA through anti-inflammation and antioxidant. Consequently, this work analyzed AKG’s role and mechanisms in mice with AAA.

This work focused on determining how AKG affected elastase-mediated AAA occurrence within mice. Here, we demonstrate that AKG treatment significantly inhibited AAA formation in this model. Apart from that, AKG supplement significantly reduced oxidative stress, macrophage infiltration in the aortic walls and inflammatory cytokines expression. Besides, we also show that AKG ameliorates AAA though PXDN/HCLO/ERK signaling pathways in vitro and in vivo.

## Methods

### Animals and mouse model

Our study protocols gained approval from the Animal Care and Use Committee of the Affiliated Hospital of Qingdao University. The C57BL/6 male mice aged 8–10 weeks were utilized for all experiments. To induce AAA, each male mouse was intraperitoneally injected with sodium pentobarbital for anesthesia, afterwards, pancreatic elastase (1.5 U) was applied locally into abdominal aorta. For exploring AKG’s efficacy in treating elastase-mediated AAA, all animals were randomized as sham (Sham), AAA, low-(0.1%, 100 mg/kg/d) and high-dose AKG 0.5% (500 mg/kg/d) groups. Accordingly, each mouse was given AKG (Macklin, China) dissolved in water and raised under the environmentally-controlled conditions for 28 consecutive days.

### Cell culture, treatment and transfection

This work acquired mouse aortic smooth muscle cells (MOVAS) in American Type Culture Collection (ATCC). For establishing the aneurysmal microenvironment within MOVAS, this work adopted TNF-α for stimulating MOVAS [15, 16]. The MOVAS cell line was grown in DMEM media that contained 1% penicillin/streptomycin and 10% fetal bovine serum (FBS), followed by incubation under 5% CO2 and 37 °C conditions. Before experiment, this work rinsed the MOVAS thrice with PBS, followed by pretreatment using AKG (5, 10, 20 mmol/L) in serum-free medium for two hour before TNF-α stimulation (100 ng/mL). Then, this work inoculated MOVAS at 50–70% confluency into the 6-well plates, followed by 6-h infection using the lentivirus expressing PXDN or lentiviral vector (MOI = 50) in serum-free medium. Afterwards, MOVAS were further cultured for a 24-h period in serum-depleted medium before TNF-α stimulation for the indicated times.

### Elisa assay

The MOVAS cells were homogenized with PBS at 4 °C and centrifuged at 13,000 rpm. Then, the levels of MCP-1, IL-1β, and IL-6 were measured using ELISA kits (abcam) strictly following instructions.

### MOVAS migration assay

For confirming cell migration, this work conducted Transwell assays. Briefly, the present work seeded MOVAS into the serum-free DMEM added into the upper Transwell chamber in the 24-well plates; whereas medium that contained 10% FBS was added into bottom chamber. At 24-h later, PBS was used to rinse cells on upper membrane surface, while those penetrating bottom chamber were subject to 4% formalin fixation as well as 1% crystal violet staining. The migrating cells were then counted microscopically for quantification. Also, cell migration was analyzed through scratch assay. In brief, MOVAS were inoculated in the 6-well plates and they attained 70–80% confluency at 24-h later. Cells were cultured for another 2-h period in serum-depleted medium prior to experiment, followed by stimulation with TNF-α and treatment with AKG for another 24 h in 0.1% FBS medium. Afterwards, one sterile micropipette tip was utilized to make scratch wounds on cell layer in every plate. Then, those detached cells were eliminated by PBS flushing, and cell proliferation was suppressed by replacing the original medium by serum-free medium, followed by TNF-α treatment of cells. After acquisition of images under the microscope at 0/24 h, the Image-Pro Plus (version 6.0) was adopted for evaluating recovered area proportion.

### Detection of ROS generation in MOVAS and in mice

This study adopted fluorescence probes DCFH-DA and DHE for measuring intracellular ROS levels within abdominal aortic tissues or MOVAS. In brief, this work cultured tissues or cells for a 40-min period using 10 μM and DCFH-DA or 10 μM DHE within the humid incubator in dark. After obtaining the ROS fluorescence intensity, this study adopted Image-Pro Plus software for quantification.

### Apoptosis assay in vivo and vitro

The present work adopted CellEvent Caspase-3/7 Green etection Reagent (Roche Diagnostics) for measuring caspase-3/7 activities in line with specific protocols. MOVAS were seeded into 48-well plates. Before experiment, MOVAS were cultured in serum-depleted medium for a 2-h period, followed by TNF-α stimulation and followed treated with AKG for another 24 h in 0.1% FBS medium. After images were captured microscopically, the Image-Pro Plus (version 6.0) was utilized for analysis.

Apoptosis of vascular cells in aortic wall was detected by TUNEL assay in line with specific instructions. Briefly, 7-μm aortic tissue sections were subject to 10-min permeabilization using 20 μg/mL Proteinase K solution under ambient temperature, followed by 10-min incubation using equilibration buffer under ambient temperature. Thereafter, aortic tissues were further incubated with terminal deoxynucleotidyl transferase (TdT) reaction mixture under 37 °C for a 60-min period, then the reaction was terminated using 2 × SSC buffer. Each slide was mounted using ProLong Gold Antifade Mountant with DAPI (Invitrogen, Thermo Fisher Scientific, P36935). Finally, green fluorescence was measured.

### Histology, immunohistochemistry (IHC) and immunofluorescence (IF) staining

Hematoxylin–Eosin (H&E) staining was conducted for analyzing cell morphology. The dilated aortic samples within infra-renal area of mice of diverse groups were treated by Tissue-Tek O.C.T. Compound embedding within liquid nitrogen, followed by slicing in the 5-μm consecutive sections. Afterwards, these sections were subject to deparaffinage, rehydration and staining using the Verhoeff-van Gieson staining kit to assess elastin in line with specific protocols.

Immunohistochemistry (IHC) staining of CD45, CD68, and MMP-9 was performed for observing changes in aortic wall morphology as well as inflammation. Briefly, the aortic slices (5 μm thickness) were later subject to 4% paraformaldehyde (PFA) fixation along with paraffin embedding. CD45, CD68 and MMP-9 were used for analysis. This study subsequently measured positive cell infiltration rate through the mean nucleus quantity surrounded by positively-stained cells under the microscope.

After blocking PFA-fixed tissue sections using 10% normal donkey serum, they were further probed under 4 °C overnight with primary ntibodies anti-α-SMA. Normal goat or rabbit IgG was applied as negative controls. After several washes with PBS, secondary antibodies were further used to probe sections under ambient temperature for a 1-h period. Nuclei were stained by DAPI. After acquisition of images under the microscope, Image-Pro Plus (version 6.0) was utilized for analysis.

### RNA sequencing (RNA-seq)

To investigate the molecular mechanism underlying the regulatory effect of AKG on the development of AAA, total RNA was extracted from the aortic tissues of AAA-treated (n = 4) and AAA + AKG-treated (n = 4) mice. The mRNA library was constructed according to the manufacturer’s protocol (VAHTS Universal V6 RNA-seq Library Prep Kit for Illumina Kit (Vazyme, NR604-02). The complementary DNA libraries were sequenced using Illumina NovaSeq6000 with 2X150 running circles. In brief, 1 μg of total RNA from each sample was used for library construction. After capture and purification, mRNA was fragmented(85 °C, 6 min) into 250–450 bp and reverse transcribed. With end repair and adaptor ligation, the library was purified and underwent size selection. A total of 13 PCR cycles were used for the upcoming library amplification. Agencourt AMPure XPTM Beads (Beckman Coulter) were used for purification and sequencing was carried out on a NovaSeqTM(PE150, Illumina). Genes with expression changes of twofold compared with control samples were considered to be significantly up- or downregulated. The GO and KEGG was analysised using TopGO.

### Transduction of mice aortic segments in vivo

We examined the role of PXDN in AKG inhibition of the development of AAA by transfection with adenovirus. The experimental procedure was performed as follows [17]. In brief, the infrarenal segment of the abdominal aorta was exposed under anesthesia (2.5% isoflurane) via a midline incision, clamped just below the renal arteries, a vascular cannula introduced into the isolated vascular segment, and the aorta flushed with normal saline. For each mouse, the segment was transduced with HBAD-EGFP (50 μl, 2.51 × 10^11^ PFU/mL) or HBAD-Adeasy-m-Pxdn-Null-EGFP (50 μl, 1.26 × 10^11^ PFU/mL). After 20 min of incubation, the cannula was removed and the incision was sutured. After 2 weeks, the C57BL/6 male mice was intraperitoneally injected with sodium pentobarbital for anesthesia, afterwards, pancreatic elastase (1.5 U) was applied locally into abdominal aorta.

### Western blotting (WB) assay

This work isolated total proteins in abdominal aorta and MOVAS. Thereafter, BCA Protein Assay Kit (Beyotime) was utilized to measure protein content. Subsequently, this study separated proteins through SDS-PAGE, followed by transfer onto PVDF membranes (pore size, 0.45 μm). Primary antibodies for MMP-2 (ABclonal), MMP-9 (Abclonal), a-SMA (Abcam), SM-22α (Abcam), PXDN (Abclonal), 3-Cl-tyr (Cell Science), BAX (Cell Signaling Technology), BCL2 (Cell Signaling Technology, USA) and ERK1/2/p-ERK1/2 (Cell Signaling Technology) were used. The present study adopted the ECL protocol for detecting the antigen–antibody complex by adopting goat anti-mouse or anti-rabbit IgG secondary antibody, with GAPDH being the endogenous reference.

### RNA isolation and real-time PCR

This work utilized TRIzol reagent for isolating total RNA in MOVAS and abdominal aortic tissue. SYBR Green was used in the quantitative real-time PCR (Takara, Japan). By normalizing the samples to GAPDH, an internal reference, the samples were relatively measured.

### Statistical analysis

GraphPad Prism 8.0 was adopted for statistical analysis. Results were displayed in a form of mean ± SEM. A Student *t*-test was used for statistical comparison between two groups. One-way analysis of variance was performed for analysis of three or more groups. P < 0.05 stood for statistical significance.

## Results

### AKG treatment inhibited elastase-induced AAA formation in mice

**Fig. 1:**
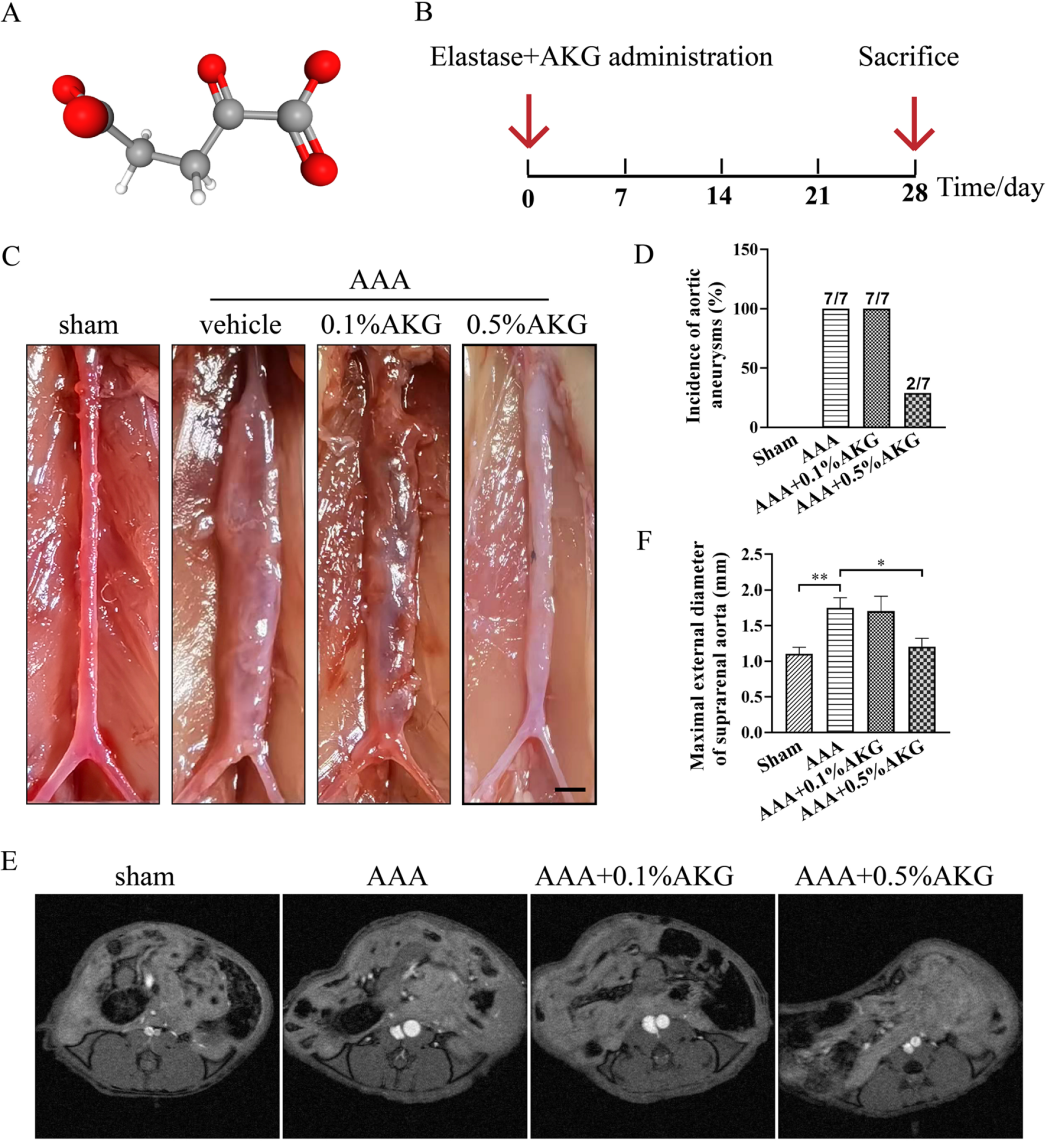
Effects of AKG treatment on elastase-induced mouse aortic aneurysms. **A** AKG's molecular structure. **B** AAA inducement and administration regimens. **C** Representative images of mouse aortic aneurysms (n = 7). **D** incidence of mouse aortic aneurysms (n = 7). **E** Representative MRI images (n = 4). **F** Maximal external diameter of suprarenal aorta (mm) (n = 7). *P < 0.05, **P < 0.01

The structure of AKG is shown in Fig. 1A. Figure 1B presented the AAA inducement and administration regimens. As displayed in Fig. 1C, D, AKG treatment significantly inhibited AAA incidence. In MRI images, AKG-treated mice exhibited markedly decreased maximum abdominal aortic diameter within the cross-sections (Fig. 1E, F). Moreover, 0.5%AKG treatment in mice with the sham surgery did not affect abdominal aortic diameter, compared with the vehicle-treated group (Additional file 1: Fig. S1). Collectively, these results supported that AKG protected mice from elastase-induced AAA formation and aortic rupture.

### AKG blocked elastin degradation, vascular remodeling and ECM degradation

**Fig. 2:**
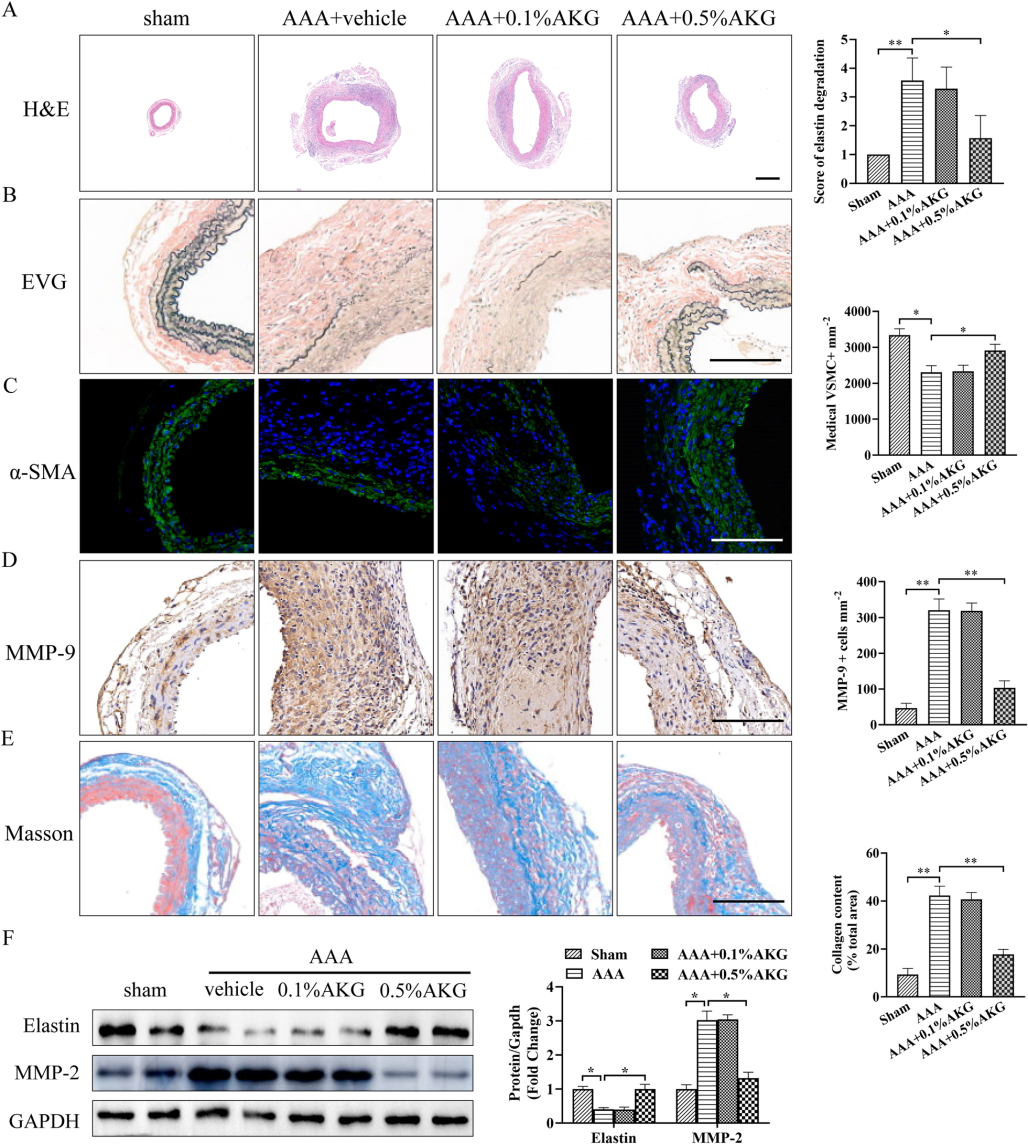
Role of AKG in elastic fibers, ECM decomposition and vascular remodeling. **A** Typical images showing HE staining for aortic cross-sections (n = 6). Bars = 200 μm. **B** Typical EVG staining as well as elastin decomposition quantification within aortic sections of specific mouse groups (n = 6). Bars = 50 μm. **C** Representative image and quantification of immunostaining for α-SMA (green). Nuclei were stained with DAPI. (n = 6). Bars = 50 µm. **D** Typical images as well as IHC staining for MMP-9 in the indicated groups (n = 6). Bars = 50 µm. **E** Representative image and quantification of Masson trichrome staining (n = 6). Bars = 50 µm. **F** The active-MMP2 (68 kDa) and elastin expression were analyzed by western blot (n = 4).*P < 0.05, **P < 0.01

We next investigated whether AKG treatment could block elastin degradation and vascular remodeling. H&E staining and EVG staining revealed high dose of AKG decreased arterial medial elastin decomposition, thereby preserving the integrity of aortic structure compared to AAA group (Fig. 2A, B). AKG-treated mice exhibited a marked reduction in the depletion of medial smooth muscle α-actin cells (Fig. 2C). Moreover, AKG treatment inhibited the expression of MMP-9 in aortic wall (Fig. 2D). Additionally, AKG-treated mice exhibited a significant decrease matrix remodeling in suprarenal aortic adventitia compared with AAA group (Fig. 2E). Moreover, 0.5%AKG treatment in mice with the sham surgery did not affect arterial medial elastin decomposition and vascular remodeling, compared with the vehicle-treated group (Additional file 1: Fig. S1). Consistently, Western blot analysis showed that AKG treatment significantly inhibited the MMP-2 expression and upregulated the elasitin expression (Fig. 2F). These results suggested that AKG was important for preventing from vascular remodeling and ECM degradation in AAA formation.

### AKG attenuated inflammation, reactive oxygen species (ROS) generation and apoptosis of vascular smooth muscle cells (VSMCs) in elastase-induced AAA formation

**Fig. 3:**
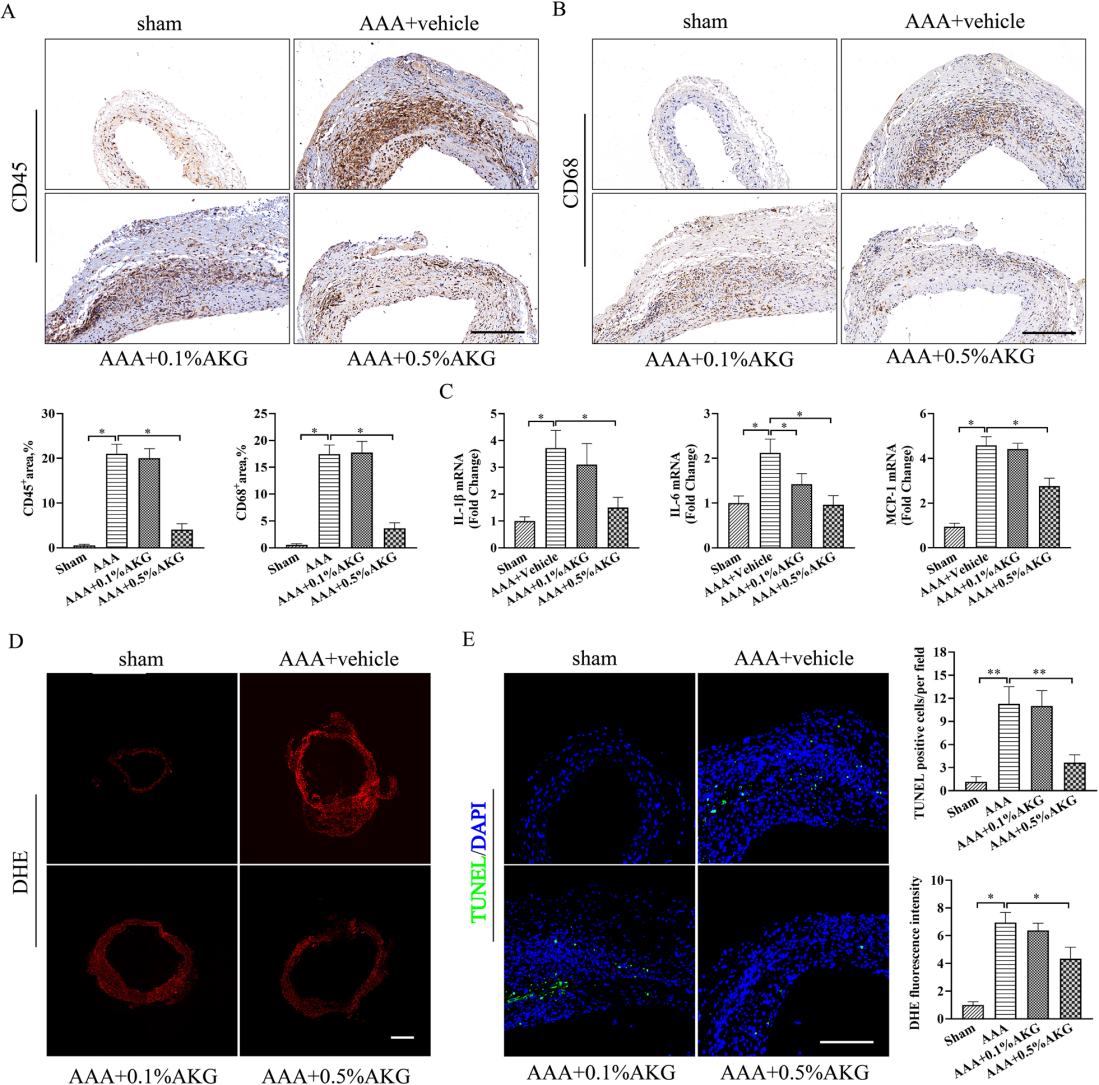
AKG attenuated inflammation, oxidative stress and apoptosis of VSMCs in elastase-induced AAA formation. **A**, **B** Typical images as well as IHC staining for CD45 and CD68 in the indicated groups (n = 6). Scale bar = 50 μm. **C** IL-1β, IL-6 and MCP-1expression was analyzed by qPCR (n = 5). **D** Typical images as well as quantification of superoxide anions detected by DHE staining. (n = 5). Scale bar = 200 μm. **E** TUNEL assay (n = 5). TUNEL, green; DAPI, blue. Scale bar = 50 μm.*P < 0.05

OS and inflammation have critical effects on AAA occurrence [4]. As anticipated, high-dose AKG treatment markedly suppressed inflammation in the vascular walls of mice as assessed by immunohistochemical staining of CD45 (leukocytes) and CD68 (macrophages) within aortic wall (Fig. 3A, B). Consistently, the increased IL-1β, IL-6 and MCP-1 levels within elastase-mediated AAA occurrence in mice were suppressed by AKG treatment compared with model group (Fig. 3C). A 14-day elastase incubation dramatically enhanced ROS within the aortic wall compared with sham group, and the AKG substantially reduced oxidative stress in elastase-induced AAA formation (Fig. 3D). Additionally, AKG treatment markedly suppressed elastase-induced VSMCs apoptosis in the aorta (Fig. 3E).

### AKG blocked the loss of VSMCs contractile phenotype induced by TNF-α and facilitated a synthetic phenotype in VSMCs

**Fig. 4:**
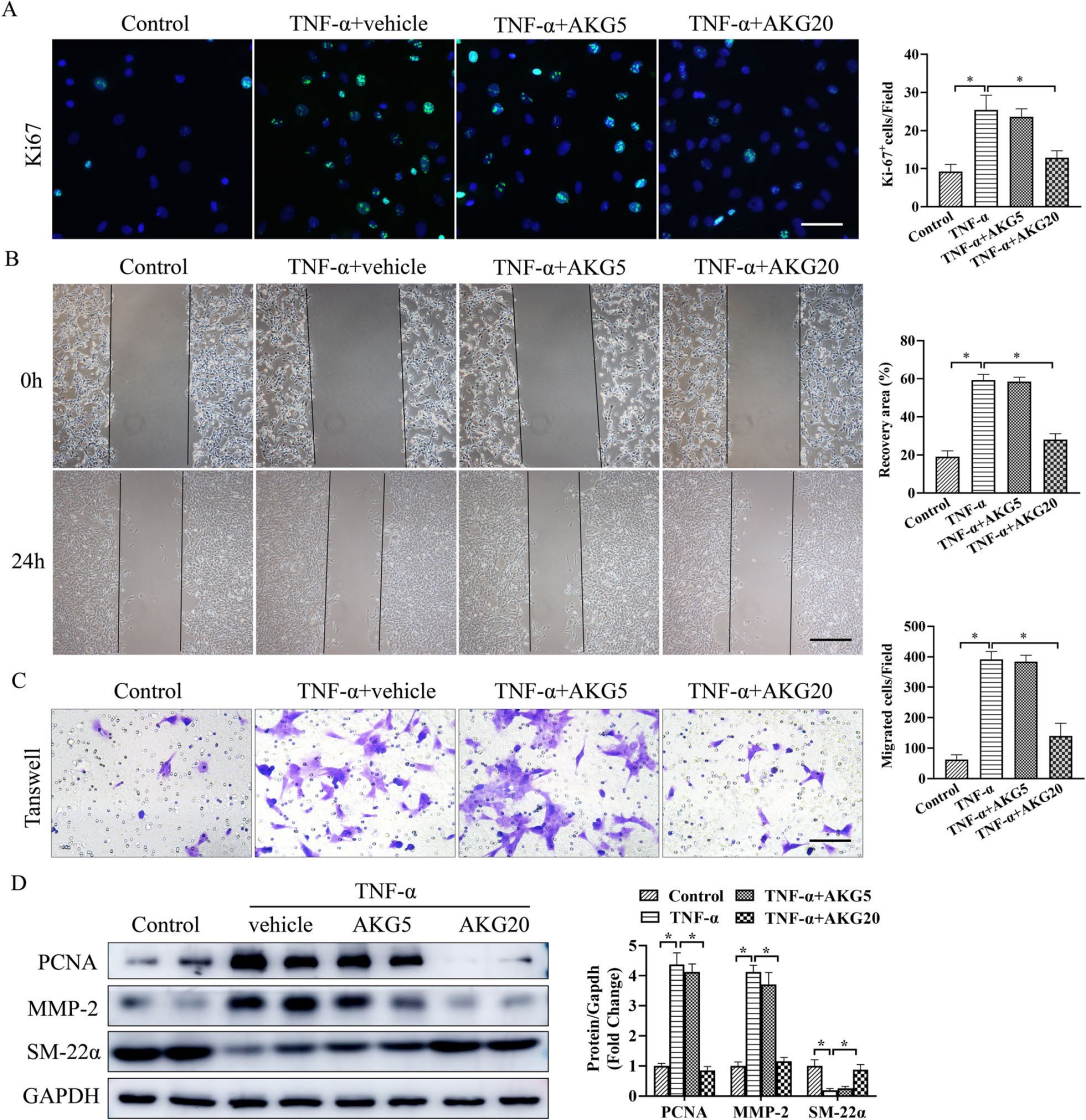
AKG prevented TNF-α-caused synthetic phenotype acquisition in vitro. **A** Representative image and quantification of VSMCs proliferation by Ki-67 staining (n = 5). **B** Typical image as well as quantitative analysis on VSMCs invasion by wound scratch assay (n = 4). **C** Typical image as well as quantitative analysis on VSMCs migration by transwell assay (n = 4). **D** The active MMP-2 (68 kDa) and SM22α expression were analyzed by western blot (n = 4). *P < 0.05

For better examining how AKG blocked AAA occurrence, this study treated VSMCs with TNF-α for mimicking the AAA microenvironment in vitro. We selected AKG concentrations of 0, 5, 10, 20, 50 or 100 mM to assess its roles on VSMCs. At 24 h, this work measured cell viability after treating with AKG (Additional file 1: Fig. S2). Transwell assays were carried out to measure AKG's role in VSMCs migration. As shown in Fig. 4A, treatment with AKG reduced VSMCs proliferation after exposure to TNF-α. Furthermore, AKG-treated VSMCs migration after TNF-α treatment was markedly reduced (Fig. 4B, C). According to WB assay, AKG exposure increased differentiated SMC marker (SM-22α) expression but reduced PCNA and MMP-2 expression within those cultivated VSMCs (Fig. 4D). These results suggested that AKG inhibited the loss of VSMCs contractile phenotype and facilitated a synthetic phenotype in VSMCs.

### AKG attenuated TNF-α-induced inflammation, oxidative stress and apoptosis in VSMCs

**Fig. 5:**
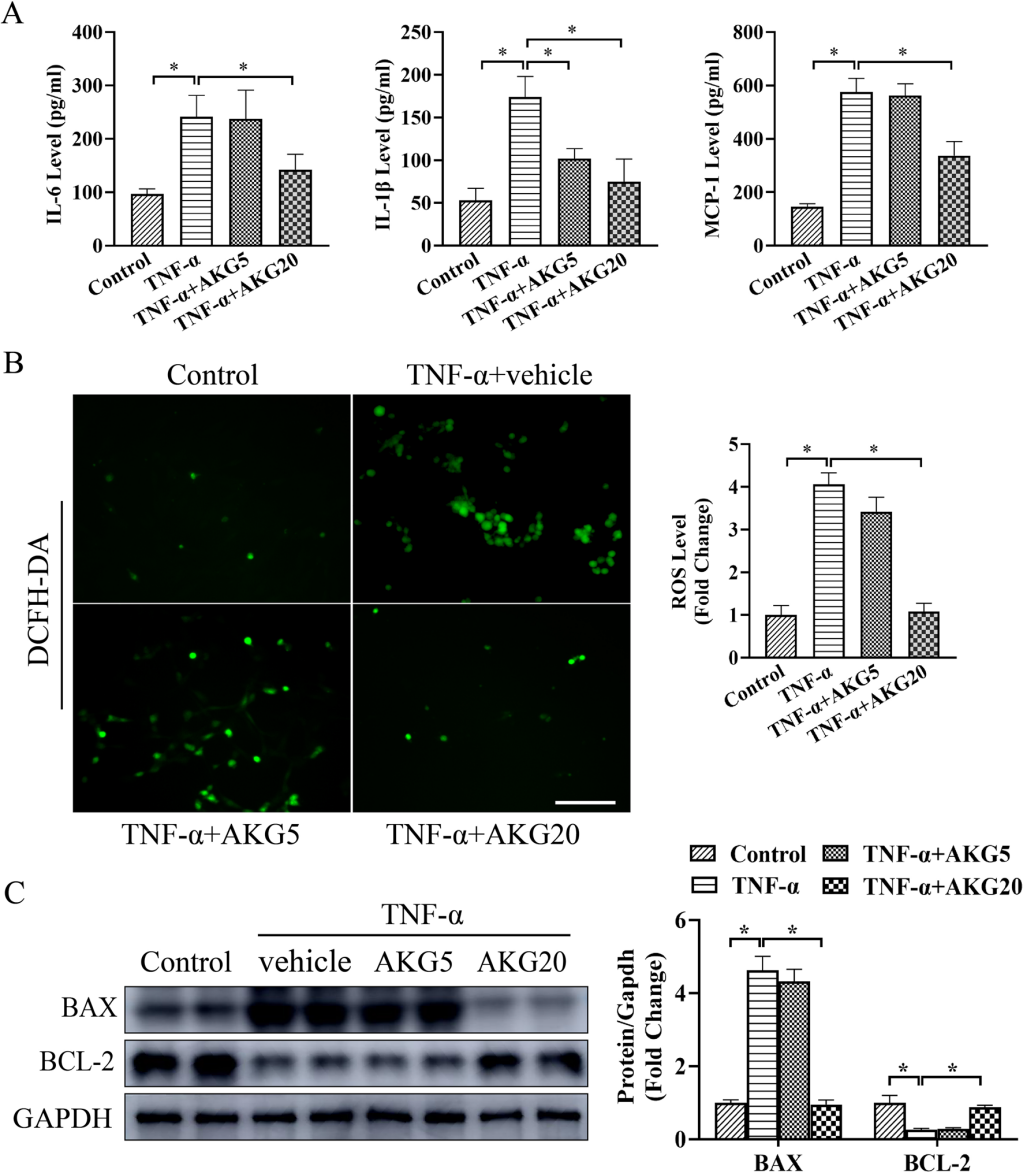
AKG suppressed TNF-α-mediated OS, apoptosis and inflammation in vitro. A MCP-1, IL-1β and IL-6 expression was measured using ELISA (n = 5). **B** Representative image and quantification of superoxide anion levels measured by DCFH-DA staining (n = 5) **C** The BAX and BCL-2 expression were examined through WB assay (n = 4). *P < 0.05, **P < 0.01

We further determine how AKG affected OS, apoptosis and inflammation of VSMCs. In TNF-α-induced VSMCs, MCP-1, IL-1β and IL-6 expression markedly elevated, as shown in Fig. 5A, and AKG significantly reversed these alterations. The production of superoxide was significantly inhibited by AKG compared to the TNF-α stimulation group (Fig. 5B). In addition, we also found that AKG treatment markedly suppressed TNF-α-induced VSMCs apoptosis, as assessed by Western blot assays (Fig. 5C). Based on the above findings, AKG suppressed TNF-α-mediated OS, apoptosis and inflammation of VSMCs.

### RNA sequencing highlighted biological processes and target molecules modulated by AKG

**Fig. 6:**
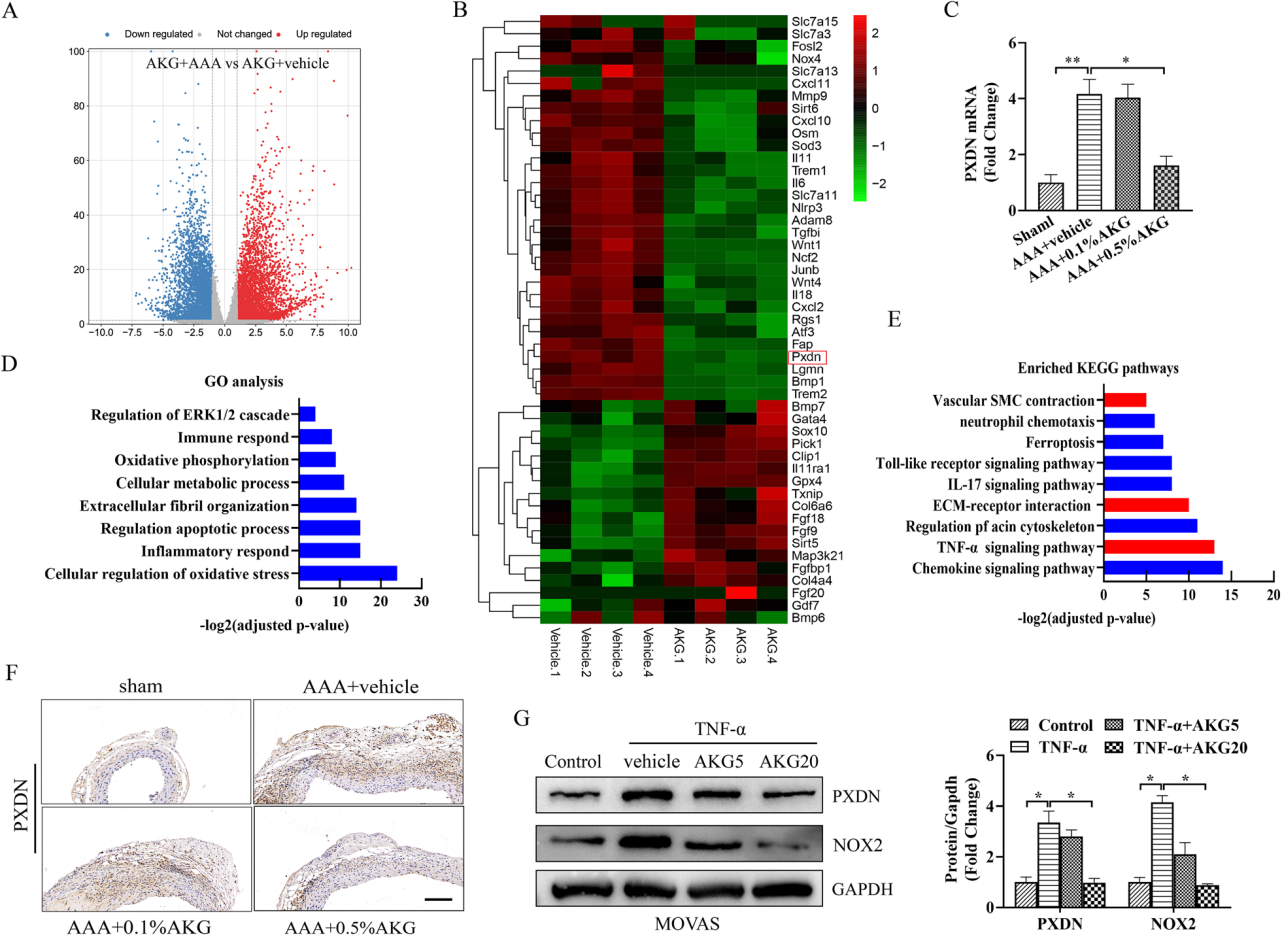
The expression of PXDN was down-regulated in AKG-treated mice. **A** Volcano plot showing different gene levels in aortic transcriptome. **B** Heat map showing levels of genes that encoded proteins having typical oxidoreductase activity along with inflammatory response. **C** RT-qPCR assay was conducted to analyze PXDN level (n = 4). **D** Gene ontology (GO) analysis. **E** KEGG pathway analysis. **F** ICH was conducted to analyze PXDN level (n = 4). **G** WB assay and quantitative analysis of PXDN level (n = 4)

For investigating the mechanism by which AKG protected against AAA formation, we performed the RNA sequencing (RNA-seq). In RNA sequencing, the significance threshold was set at adjusted P-value < 0.05 and fold changes (FC) < 0.67 or > 1.5 of gene levels across diverse groups (Fig. 6A). Subsequently, we performed functional clustering analysis and KEGG analysis among DEGs. Most of the related biological processes (BPs), like the oxidative damage, the inflammatory responses, immune response, as well as regulation of apoptotic processes, was related to AAA formation (Fig. 6D, E). As reported, inflammation and excessive ROS were identified as the major causes leading to AAA occurrence. This work discovered that genes that encoded proteins having typical inflammatory response and oxidoreductase function were down-regulated in AKG-treated mice (Fig. 6B). PXDN has been previously suggested to catalyze hypochlorous acid (HOCL) generation in HO, which also markedly increases the ROS production and the inflammatory response [15, 16]. PXDN is the new signal node mediating the phenotype change of VSMCs, which also has an important effect on AAA formation [17]. In line with prior works, this study selected PXDN gene for furter analysis. PXDN was involved in above biological processes. Moreover, the changes in PXDN expression were detected through qPCR (Fig. 6C), ICH (Fig. 6F) and WB (Fig. 6G) assays in vivo and in vitro (Fig. 6C–G). Therefore, we considered that AKG’s protection against AAA occurrence was tightly related to the reduction of PXDN expression.

### Lentivirus-mediated PXDN overexpression weakened the protective effect of AKG in vitro

**Fig. 7:**
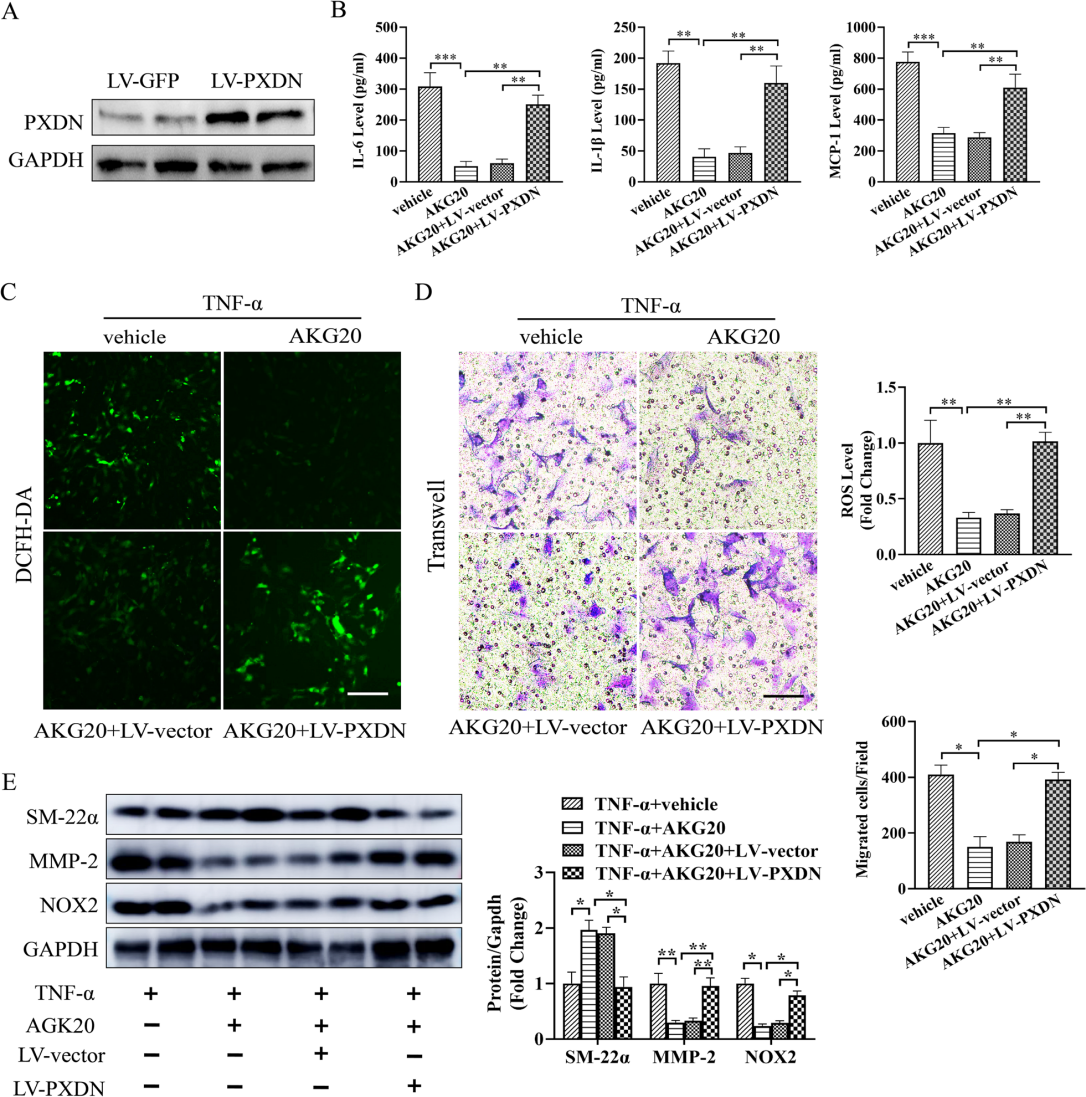
Lentivirus-mediated PXDN up-regulation reduced AKG’s protection in vitro. **A** The PXDN expression were analyzed by western blot (n = 4). **B** MCP-1, IL-1β and IL-6 expression was measured using ELISA (n = 5). **C** Typical images as well as quantitative analysis on superoxide anion expression measured through DCFH-DA staining (n = 5). **D** Typical images as well as quantitative analysis on VSMCs migration by transwell assay (n = 4). **E** The active MMP-2 (68 kDa), SM-22α and NOX2 expression were examined through WB assay (n = 4). *P < 0.05, **P < 0.01

For better evaluating PXDN’s effect on AKG-induced VSMCs regulation in vitro, VSMCs were infected with lentivirus-mediated PXDN (Fig. 7A). As a result, PXDN up-regulation suppressed AKG’s inhibition against TNF-α-mediated VSMCs inflammation (Fig. 7B), oxidative stress (Fig. 7C) and contractile phenotype (Fig. 7D) in vitro. Similarly, MMP-2, SM22α and NOX2 expression showed similar trend, as revealed by WB assays (Fig. 7E). Taken together, the above findings indicated that PXDN up-regulation markedly reduced AKG’s protection in vitro.

### PXDN overexpression weakened the protective effect of AKG in vivo

**Fig. 8:**
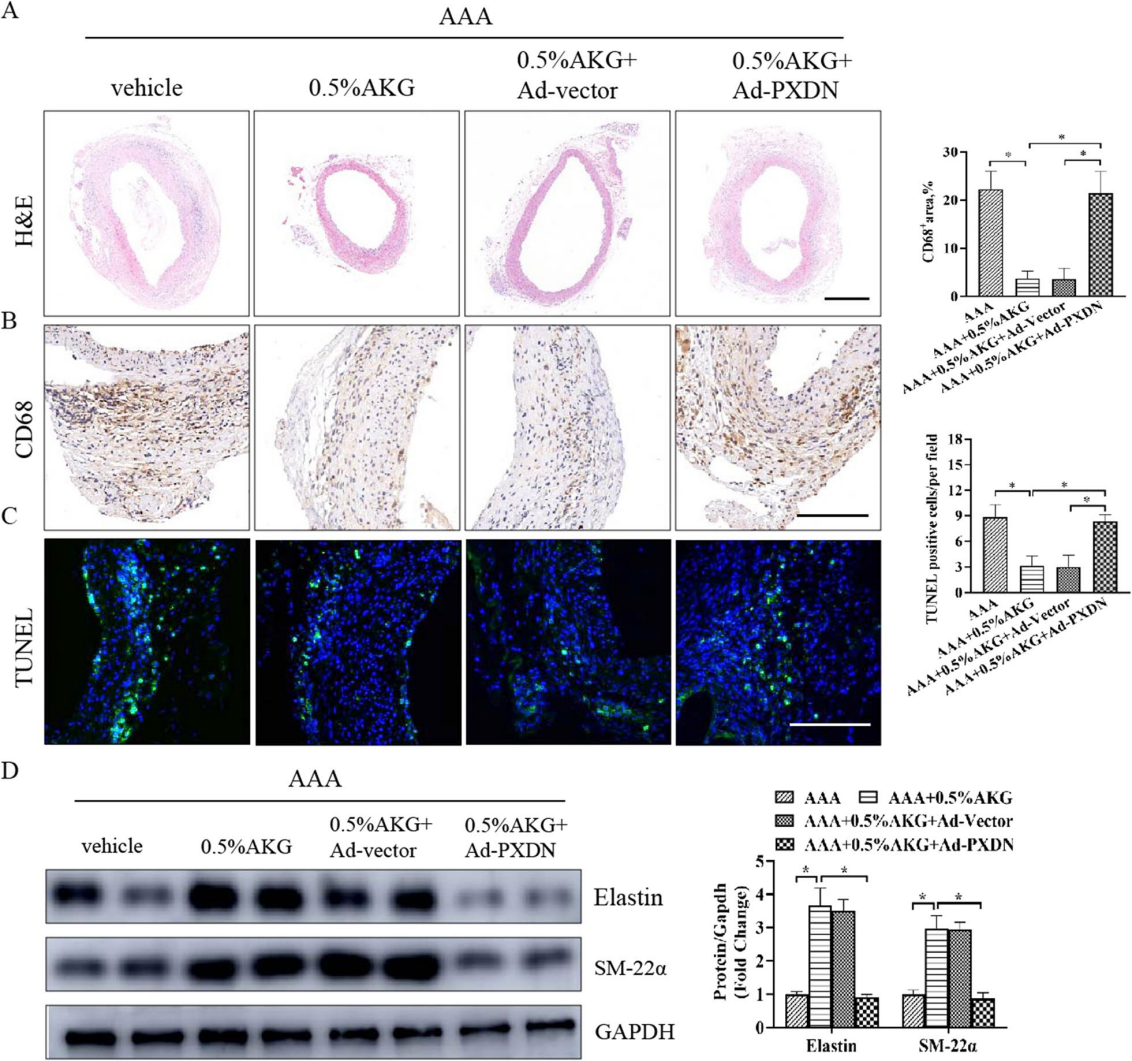
PXDN overexpression weakened the protective effect of AKG in vivo. **A** Typical images showing HE staining for aortic cross-sections (n = 6). Bars = 200 μm. **B** Typical images as well as IHC staining for CD68 in the indicated groups (n = 6). Scale bar = 50 μm. **C** TUNEL assay (n = 5). TUNEL, green; DAPI, blue. Scale bar = 50 μm. **D** The elastin and SM-22α expression were examined through WB assay (n = 4). *P < 0.05

To further evaluate whether PXDN is involved in the development of AAA, PXDN was overexpressed in aorta by the injection of a adenovirus harboring the PXDN gene (Ad-PXDN) (Additional file 1: Fig. S3). PXDN overexpression weakened the protective effect of AKG, which manifested as significant decreases in the integrity of aortic structure in AAA + 0.5%AKG + Ad-PXDN group, compared with AAA + 0.5%AKG + Ad-Vector group (Fig. 8A). Moreover, PXDN overexpression exhibited more severe inflammatory response and more cell apoptosis in the aorta (Fig. 8B, C). Similar results were also observed for the expression levels of Elastin and SM-22α in aorta (Fig. 8D). Taken together, these results suggest that PXDN overexpression weakened the protective effect of AKG in vivo.

### AKG blocked PXDN/HOCL/ERK signaling pathways in AAA formation and TNF-α stimulated VSMCs

**Fig. 9:**
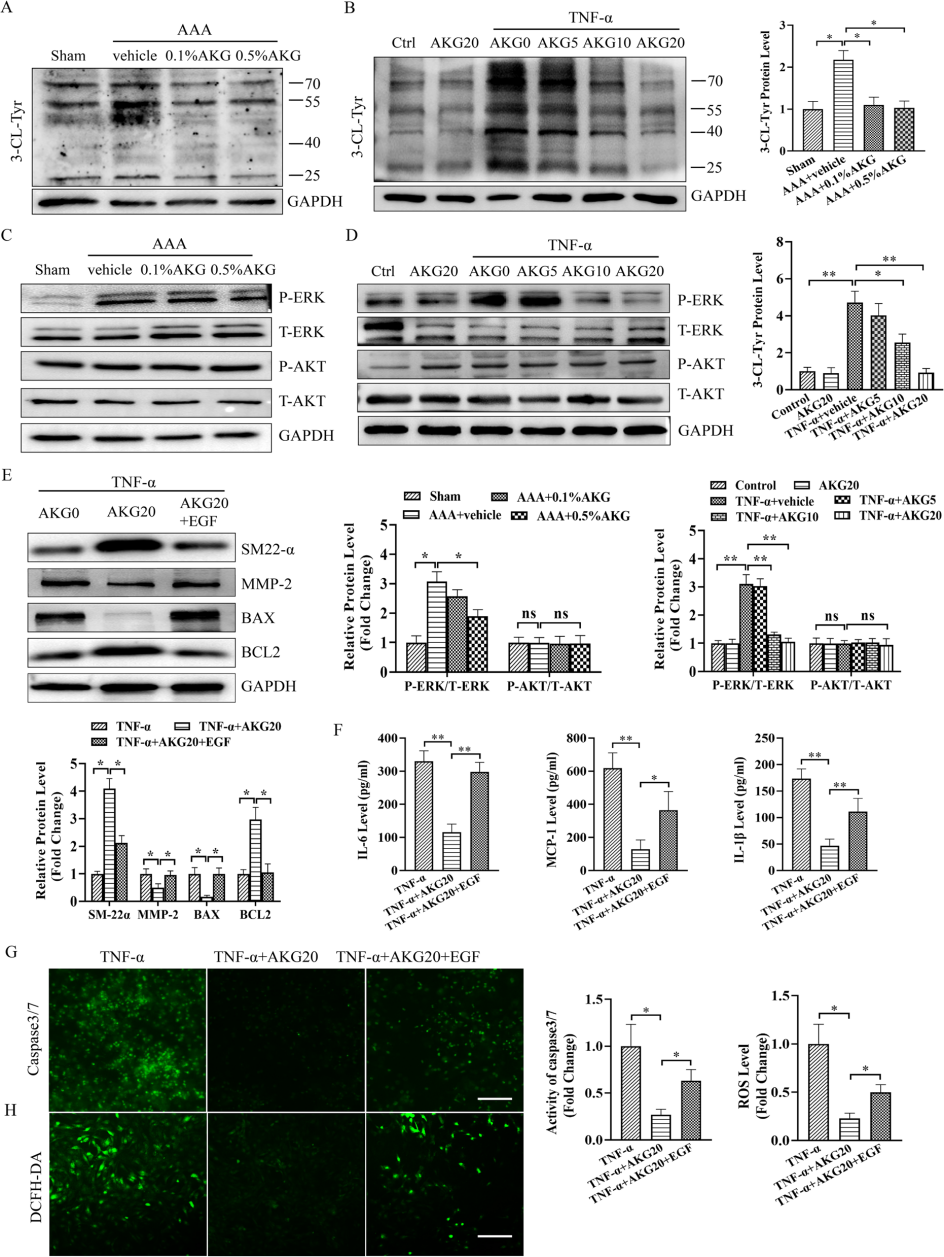
AKG blocked PXDN/HOCL/ERK pathways in vivo and in vitro. **A**–**E** 3-Cl Tyr, p-ERK, p-AKT, SM-22α, active MMP-2 (68 kDa), BAX and BCL2 levels were analyzed by western blot (n = 4). **F** MCP-1, IL-1β and IL-6 expression was measured using ELISA (n = 5). **G** Caspase 3/7 activities were measured to analyze MOVAS apoptosis (n = 5). **H** Representative image and quantification of superoxide anion expression measured through DCFH-DA staining (n = 5). *P < 0.05, **P < 0.01

**Fig. 10:**
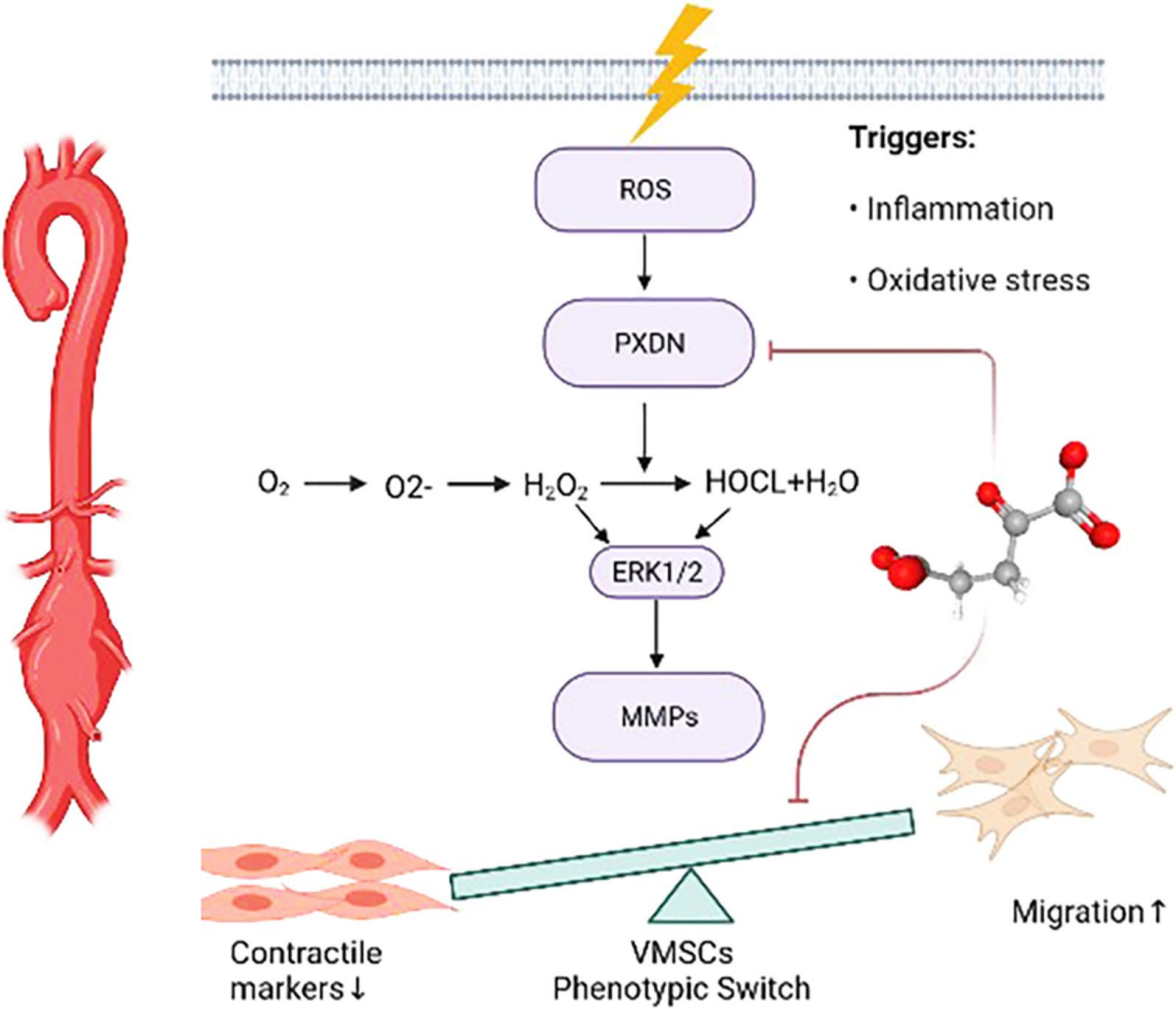
Model illustrating that AKG-mediated inhibition of PXDN/HOCL/ERK signaling pathways in abdominal aortic aneurysm

Previous studies have shown that PXDN shows major expression within cardiovascular system, which also plays an important role in HOCL generation [18, 19]. PXDN promoted VSMC phenotypic switch through the HOCL/ERK 1/2 signaling [18, 20]. The present work evaluated AKG's function in 3-Cl Tyr, p-ERK and p-AKT expression in AAA formation and TNF-α stimulated VSMCs by western blot. As a result, AKG inhibited p-ERK1/2, 3-Cl Tyr levels but not p-AKT in vivo and in vitro (Fig. 9A–D). Furthermore, ERK activator disrupted the protective of AKG on TNF-α-mediated apoptosis, OS, inflammation and contractile phenotype of VSMCs, in vitro (Fig. 9E–H). The obtained results suggested that AKG ameliorated abdominal aortic aneurysm via inhibiting PXDN/HOCL/ERK1/2 signaling pathways (see Fig. 10).

## Discussion

AKG is an important intermediate produced during Krebs cycle, and it was originally identified as an antioxidant [21]. It was involved in different metabolic and cellular pathways, including the oxidation of fatty acids, nitrogen and ammonia balance and energy metabolism [22]. Thereafter, AKG is identified with several therapeutic activities, such as enhancing lifespan, protecting age-related osteoporosis, enhancing muscle strength and endurance, and managing non-alcoholic fatty liver disease (NAFLD) [11, 23, 24]. AKG was beneficial for cell apoptosis, OS and inflammation, which indicates that it may be applied in treating cardiovascular diseases (CVDs) too [25]. Nonetheless, the roles of AKG in AAA was previously unknown. In this study, AKG significantly suppressed the aneurysmal dilation by attenuating the oxidative stress, macrophage infiltration, elastin degradation and collagen fibers remodeling.

Previous studies have shown that AAA represents the degenerative vascular complication, with the pathological characteristics of depleted VSMCs, dysregulated ROS, infiltration of inflammatory cells and ECM degradation [7, 8]. The production of ROS and oxidative stress is thought to be a critical mechanism implicated in AAA formation because these have been shown to promote inflammation, matrix degradation, and apoptosis of VSMCs in AAA formation [26]. ROS, including hydroxyl radical (•OH), superoxide (O2^−^) along with hydrogen peroxide (H2O2), are the highly reactive chemical molecules derived from oxygen [27]. Excessive ROS levels implicated in VSMCs dysfunction, DNA damage, and lipid/protein peroxidation may result in cellular injury and death irreversibly [28]. In the development of AAA, the large numbers of infiltrating macrophages can produce large amounts of O2^−^ and H_2_O_2_ through membrane-bound NADPH oxidase. In addition, endothelial cells, VMSCs, and fibroblasts are capable of forming O2^−^ via several pathways [7]. As an antioxidant, AKG is adopted for treating some disorders, including inhibiting cardiac remodeling, suppressing colorectal cancer and reducing chronic renal failure. These may be related to regulating the activity of hypoxia, AMPK/mTOR and Wnt pathways [9, 12, 21]. As far as we know, the present work is the first to analyze AKG’s role in AAA occurrence. As a result, AKG dramatically inhibited inflammatory factor levels within vascular walls (like TNF-α, IL-6, IL-1β) and triggered a dramatic decrease of ROS compared with sham group. In vitro, AKG treatment markedly suppressed TNF-α-induced VSMCs apoptosis and the production of superoxide. For investigating the mechanism by which AKG affected AAA occurrence, RNA-seq was conducted to comprehensively analyze whole-genome gene expression profiles within the abdominal aorta in AAA group and AKG-treated group. Consistent with previous studies, numerous related BPs, such as inflammatory response, cell response to OS, immune regulation and cellular energy and metabolism, were found. Finally, cells were treated with NAC for inhibiting AAA in vitro, aiming to investigate the necessity of ROS for AKG’s protection, and AKG could not further augment its improvements.

As a result, PXDN showed marked down-regulation within AKG group compared with AAA group by RNA-seq analysis. PXDN, which belongs to hemecontaining peroxidase family, shows high expression within cardiovascular system. Previous studies have shown that PXDN promoted the cardiovascular oxidative damage, including cardiac fibrosis after MI, myocardial ischemia–reperfusion injury and hypertresion [29, 30]. A study by Huihui Peng showed that PXDN expression increased within aneurysmal tissues in mice and humans relative to healthy counterparts. PXDN catalyzed hypochlorous acid formation via H_2_O_2_, while markedly promoting the production of ROS [20]. We confirmed that AKG inhibited PXDN expression in the vascular tissues and VMSCs. Collectively, we found that PXDN modulates MOVAS phenotypic switch whereas PXDN up-regulation reduced AKG’s protection in vitro. This work also discovered that AKG dramatically inhibited -Cl-tyr level in vitro and in vivo (which was produced by the reaction between HOCl and tyrosine residues). HOCl accounts for a potent ROS family oxidant and aggravates oxidative stress in AAA formation. PXDN overexpression blocked the role of AKG on inhibiting the production of HOCl. These results demonstrated that PXDN regulates the phenotype change of VSMCs via VPO1/HOCl pathway, and it possibly has an important function during AAA occurrence.

As a key biological compound, AKG exerted critical roles in different metabolic and cellular pathways, such as AKT /mTOR signaling, NF-kB pathway, Wnt signaling and AMPK signaling. However, whether other signal pathway is regulated by AKG on AAA formation remains unknown. It has been shown recently that PXDN promoted ROS production through ERK1/2 and AKT signaling in cardiovascular system [18, 20]. Considering that PXDN is an important target for AKG to inhibit AAA, it was speculated that the AKG-regulated phenotype change of VSMCs was possibly associated with AKT and ERK1/2 pathways. According to our results, AKG inhibited the expression of p-ERK1/2 but not p-AKT whereas ERK activator blocked the effects. Moreover, ERK activator disrupted the protective of AKG on TNF-α-induced cells apoptosis and oxidative stress.

ROS levels elevate within AAA, and this enhances the inflammation, apoptosis and ECM degradation of VSMCs. Decreasing ROS within VSMCs alleviates AAA. In this work, we found that AKG supplementation exerted an anti-oxidative stress in the development of AAA formation. AKG inhibited ROS production and prevented the progression of AAA. Mechanistically, AKG reduced ROS generation by suppressing PXDN within MOVAS depending on HOCL/ERK1/2 pathway. According to our results, AKG alleviated AAA occurrence by its antioxidation within MOVAS. The findings will provide certain foundation for developing new drugs to treat AAA, and contribute to applying AKG clinically to prevent and treat AAA.

## Supplementary Information

**Additional file 1.** AKG treatment in mice with the sham surgery did not affect abdominal aortic diameter.

**Additional file 2.** The cell viability after treating with AKG.

**Additional file 3.** PXDN was overexpressed in aorta by the injection of a adenovirus harboring the PXDN gene.

## Data Availability

All data generated in this study would be provided by the corresponding author upon request.

## References

[1] Golledge J (2019). Abdominal aortic aneurysm: update on pathogenesis and medical treatments. Nat Rev Cardiol.

[2] Pinard A, Jones GT, Milewicz DM (2019). Genetics of thoracic and abdominal aortic diseases. Circ Res.

[3] Davis FM, Daugherty A, Lu HS (2019). Updates of recent aortic aneurysm research. Arterioscler Thromb Vasc Biol.

[4] MA3RS Study Investigators (2017). Aortic wall inflammation predicts abdominal aortic aneurysm expansion, rupture, and need for surgical repair. Circulation.

[5] Brangsch J, Reimann C, Kaufmann JO, Adams LC, Onthank DC, Thöne-Reineke C, Robinson SP, Buchholz R, Karst U, Botnar RM, Hamm B, Makowski MR (2019). Concurrent molecular magnetic resonance imaging of inflammatory activity and extracellular matrix degradation for the prediction of aneurysm rupture. Circ Cardiovasc Imaging.

[6] Quintana RA, Taylor WR (2019). Cellular mechanisms of aortic aneurysm formation. Circ Res.

[7] Malecki C, Hambly BD, Jeremy RW, Robertson EN (2020). The role of inflammation and myeloperoxidase-related oxidative stress in the pathogenesis of genetically triggered thoracic aortic aneurysms. Int J Mol Sci.

[8] McCormick ML, Gavrila D, Weintraub NL (2007). Role of oxidative stress in the pathogenesis of abdominal aortic aneurysms. Arterioscler Thromb Vasc Biol.

[9] An D, Zeng Q, Zhang P, Ma Z, Zhang H, Liu Z, Li J, Ren H, Xu D (2021). Alpha-ketoglutarate ameliorates pressure overload-induced chronic cardiac dysfunction in mice. Redox Biol.

[10] Tian Q, Zhao J, Yang Q, Wang B, Deavila JM, Zhu MJ, Du M (2020). Dietary alpha-ketoglutarate promotes beige adipogenesis and prevents obesity in middle-aged mice. Aging Cell.

[11] Asadi Shahmirzadi A, Edgar D, Liao CY, Hsu YM, Lucanic M, Asadi Shahmirzadi A, Wiley CD, Gan G, Kim DE, Kasler HG, Kuehnemann C, Kaplowitz B, Bhaumik D, Riley RR, Kennedy BK, Lithgow GJ (2020). Alpha-ketoglutarate, an endogenous metabolite, extends lifespan and compresses morbidity in aging mice. Cell Metab.

[12] Tran TQ, Hanse EA, Habowski AN, Li H, Gabra MBI, Yang Y, Lowman XH, Ooi AM, Liao SY, Edwards RA, Waterman ML, Kong M (2020). α-Ketoglutarate attenuates Wnt signaling and drives differentiation in colorectal cancer. Nat Cancer..

[13] He L, Wu J, Tang W, Zhou X, Lin Q, Luo F, Yin Y, Li T (2018). Prevention of oxidative stress by α-Ketoglutarate via activation of CAR signaling and modulation of the expression of key antioxidant-associated targets in vivo and in vitro. J Agric Food Chem.

[14] Kjellman U, Björk K, Ekroth R, Karlsson H, Jagenburg R, Nilsson F, Svensson G, Wernerman J (1995). Alpha-ketoglutarate for myocardial protection in heart surgery. Lancet.

[15] Xu T, Wang S, Li X, Li X, Qu K, Tong H, Zhang R, Bai S, Fan J (2021). Lithium chloride represses abdominal aortic aneurysm via regulating GSK3β/SIRT1/NF-κB signaling pathway. Free Radic Biol Med.

[16] Fu H, Shen QR, Zhao Y, Ni M, Zhou CC, Chen JK, Chi C, Li DJ, Liang G, Shen FM (2022). Activating α7nAChR ameliorates abdominal aortic aneurysm through inhibiting pyroptosis mediated by NLRP3 inflammasome. Acta Pharmacol Sin.

[17] Chen S, Kapturczak M, Loiler SA, Zolotukhin S, Glushakova OY, Madsen KM, Samulski RJ, Hauswirth WW, Campbell-Thompson M, Berns KI, Flotte TR, Atkinson MA, Tisher CC, Agarwal A (2005). Efficient transduction of vascular endothelial cells with recombinant adeno-associated virus serotype 1 and 5 vectors. Hum Gene Ther.

[18] Cao J, Zhang G, Liu Z, Xu Q, Li C, Cheng G, Shi R (2021). Peroxidasin promotes diabetic vascular endothelial dysfunction induced by advanced glycation end products via NOX2/HOCL/Akt/eNOS pathway. Redox Biol.

[19] Colon S, Luan H, Liu Y, Meyer C, Gewin L, Bhave G (2019). Peroxidasin and eosinophil peroxidase, but not myeloperoxidase, contribute to renal fibrosis in the murine unilateral ureteral obstruction model. Am J Physiol Renal Physiol.

[20] Peng H, Zhang K, Liu Z, Xu Q, You B, Li C, Cao J, Zhou H, Li X, Chen J, Cheng G, Shi R, Zhang G (2018). VPO1 modulates vascular smooth muscle cell phenotypic switch by activating extracellular signal-regulated kinase 1/2 (ERK1/2) in abdominal aortic aneurysms. J Am Heart Assoc.

[21] Bayliak MM, Lushchak VI (2021). Pleiotropic effects of alpha-ketoglutarate as a potential anti-ageing agent. Ageing Res Rev.

[22] He L, Xu Z, Yao K, Wu G, Yin Y, Nyachoti CM, Kim SW (2015). The physiological basis and nutritional function of alpha-ketoglutarate. Curr Protein Pept Sci.

[23] Wang Y, Deng P, Liu Y, Wu Y, Chen Y, Guo Y, Zhang S, Zheng X, Zhou L, Liu W, Li Q, Lin W, Qi X, Ou G, Wang C, Yuan Q (2020). Alpha-ketoglutarate ameliorates age-related osteoporosis via regulating histone methylations. Nat Commun.

[24] Aragonès G, Auguet T, Berlanga A, Guiu-Jurado E, Martinez S, Armengol S, Sabench F, Ras R, Hernandez M, Aguilar C, Colom J, Sirvent JJ, Del Castillo D, Richart C (2016). Increased circulating levels of alpha-ketoglutarate in morbidly obese women with non-alcoholic fatty liver disease. PLoS ONE.

[25] Cai X, Yuan Y, Liao Z, Xing K, Zhu C, Xu Y, Yu L, Wang L, Wang S, Zhu X, Gao P, Zhang Y, Jiang Q, Xu P, Shu G (2018). α-Ketoglutarate prevents skeletal muscle protein degradation and muscle atrophy through PHD3/ADRB2 pathway. FASEB J.

[26] Emeto TI, Moxon JV, Au M, Golledge J (2016). Oxidative stress and abdominal aortic aneurysm: potential treatment targets. Clin Sci (Lond).

[27] Petrucci G, Rizzi A, Hatem D, Tosti G, Rocca B, Pitocco D (2022). Role of oxidative stress in the pathogenesis of atherothrombotic diseases. Antioxidants (Basel).

[28] Ushio-Fukai M, Ash D, Nagarkoti S, Belin de Chantemèle EJ, Fulton DJR, Fukai T (2021). Interplay between reactive oxygen/reactive nitrogen species and metabolism in vascular biology and disease. Antioxid Redox Signal.

[29] Ge L, Zhang G, You B, Cheng G, Chen L, Shi R (2017). The role of losartan in preventing vascular remodeling in spontaneously hypertensive rats by inhibition of the H2O2/VPO1/HOCl/MMPs pathway. Biochem Biophys Res Commun.

[30] Liu Z, Xu Q, Yang Q, Cao J, Wu C, Peng H, Zhang X, Chen J, Cheng G, Wu Y, Shi R, Zhang G (2019). Vascular peroxidase 1 is a novel regulator of cardiac fibrosis after myocardial infarction. Redox Biol.

