# Supplementary figures and images for: Alpha-ketoglutarate ameliorates abdominal aortic aneurysm via inhibiting PXDN/HOCL/ERK signaling pathways
Source: J Transl Med. 2022 Oct 8;20:461. doi: 10.1186/s12967-022-03659-2 (PMC9548204; doi:10.1186/s12967-022-03659-2)

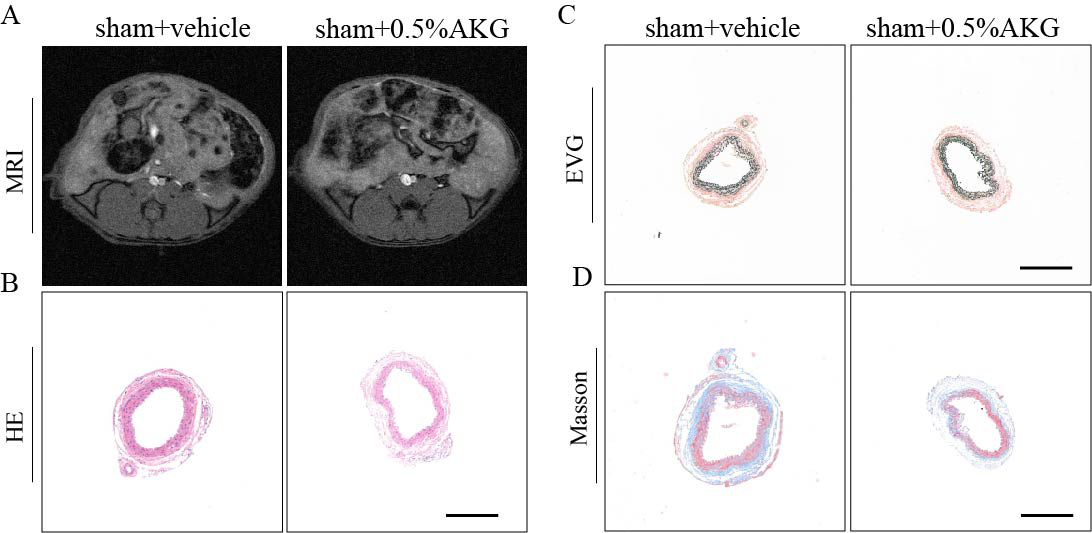

Supplement: Supplementary file 1 — Additional file 1. AKG treatment in mice with the sham surgery did not affect abdominal aortic diameter. [file 12967_2022_3659_MOESM1_ESM.jpg]

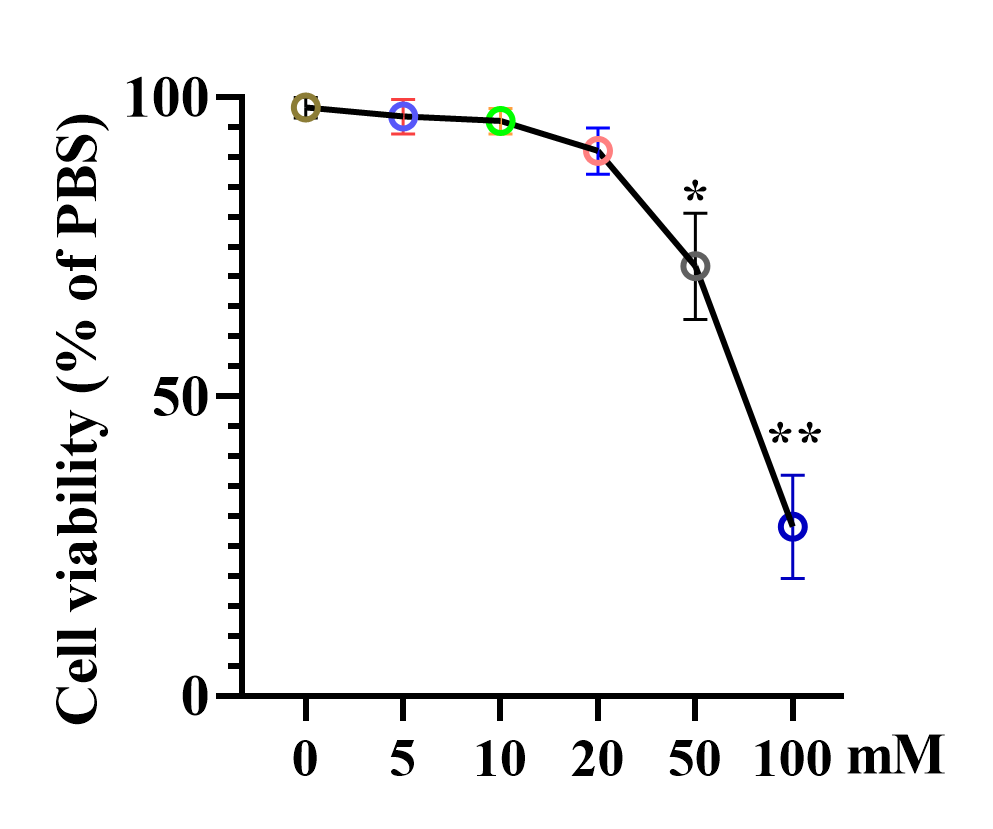

Supplement: Supplementary file 2 — Additional file 2. The cell viability after treating with AKG. [file 12967_2022_3659_MOESM2_ESM.tif]

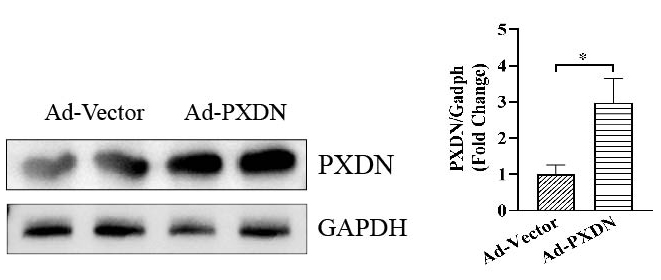

Supplement: Supplementary file 3 — Additional file 3. PXDN was overexpressed in aorta by the injection of a adenovirus harboring the PXDN gene. [file 12967_2022_3659_MOESM3_ESM.jpg]
